# Infectious disease outcomes in end-stage renal disease: the influence of dialysis type on patient vulnerability in Hail region

**DOI:** 10.3389/fmed.2026.1823284

**Published:** 2026-06-25

**Authors:** Rihab Akasha, Naif K. Binsaleh, Randa Abdeen Husien Abdalla, Obey Suliman, Ibrahim Nasser Abuqurayn, Sultan Alouffi

**Affiliations:** 1 Department of Medical Laboratory Science, College of Applied Medical Sciences, University of Hail, Hail, Saudi Arabia; 2 Medical and Diagnostic Research Centre, University of Hail, Hail, Saudi Arabia; 3 Nephrology Consultant, King Khalid Hospital, Hail, Saudi Arabia; 4 Nephrology Department Supervisor, Hail Cluster, Hail, Saudi Arabia

**Keywords:** catheter-related bloodstream infection, dialysis outcomes, hemodialysis, infection, peritoneal dialysis, peritonitis

## Abstract

**Background:**

Infections are a major threat to patients with end-stage renal disease (ESRD) receiving renal replacement therapy. Hemodialysis (HD) and peritoneal dialysis (PD) differ in access type, exposure to healthcare environments, and infection pathways, leading to distinct risks for catheter-related bloodstream infection (CRBSI), peritonitis, hospitalization, and adverse outcomes. Recognizing local patterns is essential for prevention planning and quality improvement.

**Aim:**

To compare dialysis-related infection prevalence, microbiological profiles, hospitalization events, and biochemical factors between HD and PD patients over a 3-year period (2023–2025) in a predominantly Saudi tertiary dialysis cohort, while describing overall documented infections and non-dialysis infection subtypes as secondary outcomes, and to identify potential infection risk factors relevant to local dialysis practice.

**Results:**

HD patients were older and had higher rates of diabetes and hypertension. Infection frequency was 18% in HD and 9% in PD. HD patients had higher incident rates of bloodstream infection, most of them catheter-related, whereas PD patients had higher peritonitis incidence. Infection-related hospitalization was higher in HD (12% vs. 6%), and CRP concentrations were higher in the period of infection. HD had a higher prevalence of *Staphylococcus aureus*, whereas *Staphylococcus epidermidis* was more prevalent in PD. Rates of infections decreased slightly in the study period.

**Conclusion:**

HD patients had higher infection burden and more severe outcomes compared to PD patients. Prevention strategies specific to the modality, high-profile monitoring and high-performance quality improvement programs targeted to specific dialysis populations are required to reduce the complications arising from infection.

## Introduction

End-stage renal disease (ESRD) is a major global health burden, and many affected patients require long-term renal replacement therapy through hemodialysis (HD) or peritoneal dialysis (PD). Despite advances in dialysis technology, infection remains a major contributor to poor clinical outcomes among patients receiving dialysis (1). HD patients are repeatedly exposed to vascular access manipulation, extracorporeal circuits, dialysis water systems, and frequent healthcare contact, all of which increase the risk of bloodstream infection (BSI) and access-related complications (2). Catheter-related bloodstream infections (CRBSIs) are particularly serious complications among HD patients, especially when central venous catheters (CVCs) are used, although they should be interpreted within the broader spectrum of HD-associated infectious risks rather than assumed to be the most frequent complication in every setting (3). Waterborne outbreaks in HD units have also been reported, supporting the need for strict monitoring of water quality, dialysate purity, environmental cleaning, and infection-prevention practices (4). A 2025 scoping review of healthcare-associated infections emphasized that quality-improvement interventions can reduce infections in HD facilities, but adherence to prevention protocols remains inconsistent across centers (5). National surveillance data from 2012 to 2021 similarly show that BSI trends vary by vascular access type, comorbidity profile, and facility-level infection-prevention performance (6).

PD patients have a different infection profile. Infectious peritonitis is a key PD complication because it may result in hospitalization, catheter loss, technique failure, or transfer to HD (7). Recent population-based studies suggest that potentially modifiable risks for PD-related infection include exit-site care, glycemic control, patient training, and catheter duration (8). Updated ISPD guidance emphasizes standardized definitions, prevention of exit-site and tunnel infection, and timely treatment to reduce peritonitis and catheter loss (3, 9). The Centers for Disease Control and Prevention (CDC) also emphasizes surveillance, staff education, hand hygiene, catheter-care bundles, and adherence to evidence-based infection-prevention measures in dialysis settings (10). Nevertheless, infection rates remain clinically important in dialysis units, particularly in regions with high burdens of diabetes mellitus and hypertension, both of which are common causes of ESRD and may increase infection susceptibility (11).

Recent reviews and surveillance studies indicate that HD access-related infection remains a persistent problem, with CVC use consistently identified as one of the strongest modifiable risk factors for infectious complications (12). Multidisciplinary infection-prevention programs can reduce dialysis-associated infections when practical bundles are implemented consistently (13). In parallel, national datasets have shown improvements in some bloodstream-infection indicators, although dialysis patients continue to have greater infection-related vulnerability than many other chronic disease populations (14). Updated PD catheter-related infection recommendations also highlight the importance of early diagnosis and standardized management to prevent peritonitis and catheter loss (15). In Saudi Arabia, regional evidence comparing HD and PD infection patterns over several years remains limited. Therefore, this study aimed to compare infection burden, microbiology, laboratory markers, hospitalization outcomes, and risk factors among HD and PD patients managed in a tertiary dialysis service in the Hail region over a 3-year period.

## Methodology

### Study design and setting

This retrospective observational cohort study was conducted in the dialysis unit of King Khalid Hospital, a tertiary dialysis center in the Hail region of Saudi Arabia. The unit provides both HD and PD services and maintains electronic and paper-based clinical records. The study covered the period from January 2023 to December 2025. Data were obtained from the dialysis registry, electronic medical records, the laboratory information system, and the infection-control surveillance database. The analysis evaluated infection rates, microbiological patterns, hospitalization outcomes, laboratory parameters, and temporal trends among HD and PD patients.

The study included adult patients with ESRD aged 18 years or older who were receiving maintenance dialysis during the study period. A total of 267 eligible patients were included: 216 receiving HD and 51 receiving PD. Patients were included if they had received dialysis for at least 3 months before data collection, to reduce confounding related to acute dialysis initiation. Patients who transferred to another institution, switched dialysis modality during the observation period, or had incomplete key records were excluded. HD vascular access included arteriovenous fistula (AVF), arteriovenous graft (AVG), or CVC. Most HD patients received three sessions per week, each lasting approximately 3.3–4 h. A small subgroup (4%) received four HD sessions per week during periods of clinical need, such as recurrent volume overload, hyperkalemia, or inadequate clearance; this atypical frequency was retained descriptively rather than analyzed as a separate exposure. PD patients used Tenckhoff catheters and followed a standardized nightly regimen. Patient care followed local protocols aligned with international recommendations for dialysis adequacy, infection prevention, and vascular access care.

The center primarily delivered conventional maintenance HD. Hemodiafiltration was not evaluated as a separate treatment group because it was not consistently documented as a distinct exposure in the available retrospective records; therefore, no HD-versus-hemodiafiltration subgroup comparison was performed.

During the observation period, patients who permanently shifted dialysis modality (for example, from PD to HD) were excluded from the main comparative analysis to preserve modality-specific exposure classification. Temporary or clinically required vascular-access changes among HD patients were reviewed from dialysis records and classified according to the predominant access type and the access present at the time of infection when available.

Because Saudi nationals represented most participants in both dialysis groups, the interpretation and conclusions of this study were limited to the local Hail-region Saudi dialysis population and similar tertiary-center settings, rather than generalized to all international dialysis populations.

Data were extracted through a structured data collection sheet for this study. Data collection variables included:

1. Demographic and clinical characteristics
  - Age, gender, nationality.
  - The duration of ESRD and on any present dialysis modality.
  - Smoking status.
  - Major cause of ESRD (diabetes mellitus, hypertension or other condition).
  - Comorbidities diabetes, hypertension, cardiovascular disease, among others.
2. Dialysis related variables
  - Vascular Access Type (AVF, AVG, CVC) for HD.
  - PD catheter length and regimen.
  - Frequency and duration of dialysis use.
  - Access changes or complications.
3. Laboratory parameters
  Lab values were gathered at monthly intervals and consisted of:
  - Hemoglobin.
  - Urea.
  - Creatinine.
  - Sodium.
  - Potassium.
  - Uric acid.
  - HbA1c.
  - Fasting blood sugar (FBS).
  - C reactive protein (CRP).

The most recent stable values for each year were applied to this analysis.

4 Infection related variables
  Infection data were collected from the infection-control registry, microbiology reports, and dialysis-unit logs. Variables included any documented infection, dialysis-related infection, infection type, causative organism, number of infection episodes, number of hospitalizations, hospitalization due to infection, intensive care unit (ICU) admission, severe sepsis, and infection-related mortality. Dialysis-related infections were defined *a priori* as bloodstream infection for HD patients and peritonitis for PD patients. Other documented infections, such as respiratory tract infection, urinary tract infection, skin and soft-tissue infection, and other clinically recorded infections, were included only in the secondary descriptive variable ‘any infection’ and were excluded from primary dialysis-related infection analyses. BSIs required positive blood cultures with clinical correlation according to CDC/NHSN criteria. PD peritonitis required cloudy effluent with compatible symptoms and either a positive culture or elevated peritoneal fluid white blood cell count, consistent with ISPD criteria. Viral infections were not systematically evaluated in the primary analysis. The study focused on clinically documented bacterial and fungal infections captured by infection-control and microbiology records; viral screening was not uniformly available across all patients and years. Antibiotic exposure was reviewed when documented to confirm treatment of infectious episodes; however, antibiotic type, duration, timing, and prior prophylaxis were not sufficiently complete or standardized for inclusion as independent predictors in the regression model. This was therefore treated as a descriptive clinical variable and acknowledged as a limitation. Respiratory infections, including clinically diagnosed pneumonia, and urinary tract infections were considered as non-dialysis infections and were included only within the secondary descriptive category of any documented infection. They were not included in the primary dialysis-related infection endpoint unless they were accompanied by a qualifying bloodstream infection or PD peritonitis episode.

### Outcome measures

The primary outcome was the patient-level dialysis-related infection rate between HD and PD patients, defined *a priori* as bloodstream infection for HD and peritonitis for PD. Overall documented infection and non-dialysis infection subtypes were evaluated as secondary descriptive outcomes.

### Statistical analysis

Data were analyzed using IBM SPSS Statistics for Windows, version 28.0 (IBM Corp., Armonk, NY, USA). Continuous variables were tested for normality using the Shapiro–Wilk test. Normally distributed variables were reported as mean ± standard deviation and compared with the independent-samples t test. Non-normally distributed variables were reported as median and interquartile range (IQR) and compared with the Mann–Whitney U test.

Categorical variables were presented as frequencies and percentages and compared using the chi-square test or Fisher’s exact test, as appropriate. Temporal trends across study years were evaluated using the Cochran-Armitage trend test for categorical variables and repeated-measures ANOVA or the Friedman test for continuous variables, depending on distribution. Multivariable logistic regression was used to identify independent predictors of infection. Candidate predictors included age, sex, diabetes mellitus, hypertension, dialysis duration, vascular access type, CRP level, and number of comorbidities. Odds ratios (ORs) with 95% confidence intervals (CIs) were reported. Statistical significance was set at *p* < 0.05.

### Ethical considerations

This research followed the rules of the Declaration of Helsinki. Ethical approval was taken from the Institutional Review Board (IRB) of Hail Health Cluster, Hail Region, Kingdom of Saudi Arabia (IRB Log Number: 2024 107; approval date: 5 November 2024). All research was subject to IRB Full Review Approval.

Informed consent was taken for publication. Data extraction involved the removal of all identifiers to ensure confidentiality. Permission from the concerned institution was obtained before commencing data collection, and the research team followed IRB standards for continuous reporting during the study period.

## Results

From 2023 to 2025, a fixed cohort of patients with ESRD was followed longitudinally. The study included 216 patients receiving HD and 51 patients receiving PD, giving a total sample of 267 patients. HD patients received routine maintenance dialysis throughout follow-up; the study dataset represented longitudinal patient follow-up and annual clinical records rather than a count of individual dialysis sessions. The number of PD patients remained stable across the years (50 in 2023, 51 in 2024, and 51 in 2025), reflecting continuity of care rather than repeated new enrollment.

HD patients represented 80.2% of the cohort, while PD patients accounted for 19.8%. All participants were monitored consistently throughout the study period, with systematic collection of demographics, clinical, laboratory, and infection-related data.

The cohort was predominantly Saudi (92% of HD patients and 94% of PD patients), so the observed infection patterns primarily reflect the experience of Saudi dialysis patients treated at this Hail tertiary center.

### Baseline characteristics

HD patients were significantly older than PD patients (61.4 ± 14.2 vs. 48.7 ± 13.1 years, *p* < 0.001; Table 1). The proportion of females was similar between groups (HD: 58%, PD: 62%, *p* = 0.41), as was the proportion of Saudi nationality (HD: 92%, PD: 94%). Median dialysis duration was longer in HD patients (4.0 years [IQR 3–7]) compared with PD patients (3.0 years [IQR 2–5], *p* = 0.03).

**Table 1:** Baseline characteristics of the study population (HD vs. PD).

Characteristic	HD (*n* = 216)	PD (*n* = 51)	*p*-value
Age (years), mean ± SD	61.4 ± 14.2	48.7 ± 13.1	
Female (%)	58% (125)	62% (32)	0.41
Saudi nationality (%)	92% (199)	94% (48)	0.52
Duration of dialysis (years), median (IQR)	4.0 (3–7)	3.0 (2–5)	0.03
Diabetes mellitus (%)	72% (155)	48% (24)	
Hypertension (%)	88% (190)	76% (39)	0.002
Smoking (%)	9% (19)	4% (2)	0.04
Primary cause: Diabetic nephropathy	63% (136)	41% (21)	
Primary cause: Hypertensive nephrosclerosis	27% (58)	33% (17)	0.19
Primary cause: Other	10% (22)	26% (13)	—

Comorbidities were more prevalent in the HD group, including diabetes mellitus (72% vs. 48%, *p* < 0.001) and hypertension (88% vs. 76%, *p* = 0.002). Smoking was reported in 9% of HD patients and 4% of PD patients (*p* = 0.04). Diabetic nephropathy was the leading cause of ESRD in both groups but was more common in HD patients (63% vs. 41%, *p* < 0.001), followed by hypertensive nephrosclerosis and other etiologies.

### Dialysis access and treatment

Dialysis access and treatment characteristics are described in the text and incorporated into subsequent analyses. Among HD patients, AVF was the most common access type (61%), followed by CVC (35%) and AVG (4%). All PD patients used Tenckhoff catheters and followed a standardized nightly regimen. HD was performed three times per week in 96% of patients and four times per week in 4% for specific clinical indications.

### Infection profile

As shown in Table 2, dialysis-related infection rates were higher in HD patients. Any documented infection occurred in 39 HD patients (18.1%) and 5 PD patients (9.8%; *p* = 0.02). Dialysis-related infections occurred in 30 HD patients (13.9%) and 4 PD patients (7.8%; *p* = 0.03). Bloodstream infection was the predominant dialysis-related infection in HD (24 patients, 11.1%), whereas peritonitis was the defining dialysis-related infection in PD (4 patients, 7.8%; 6 total peritonitis episodes). Non-dialysis infections were uncommon and are shown explicitly by subtype in Table 2.

**Table 2:** Infection rates and infection types by dialysis modality.

Infection variable	HD (*n* = 216)	PD (*n* = 51)	*p*-value
Any documented infection (patient-level)*	39 (18.1%)	5 (9.8%)	0.02
Dialysis-related infection (primary outcome)†	30 (13.9%)	4 (7.8%)	0.03
Bloodstream infection (BSI)	24 (11.1%)	0 (0.0%)	—
Catheter-related bloodstream infection among BSI	19/24 BSI episodes (79.2%)	Not applicable	—
Peritonitis, patients affected	Not applicable	4 (7.8%)	—
Peritonitis, total episodes	Not applicable	6 episodes	—
Total non-dialysis infections (secondary)	9 (4.2%)	1 (2.0%)	NS
Pneumonia/respiratory tract infection	4 (1.9%)	0 (0.0%)	NS
Urinary tract infection	3 (1.4%)	1 (2.0%)	NS
Skin/soft-tissue infection	2 (0.9%)	0 (0.0%)	NS
Other clinically documented non-dialysis infection	0 (0.0%)	0 (0.0%)	—

*Any documented infection is reported at patient level and includes dialysis-related and non-dialysis infections. †Dialysis-related infection was defined a priori as bloodstream infection for HD and peritonitis for PD. PD peritonitis is shown both as affected patients and total episodes because some patients experienced recurrent episodes. NS = no significant difference.

Respiratory tract infection/pneumonia, urinary tract infection, and skin/soft-tissue infection were captured as secondary non-dialysis infections and were not included in the primary dialysis-related infection endpoint unless accompanied by qualifying bloodstream infection or PD peritonitis.

The microbiological profile is presented separately in Table 3. Gram-positive organisms accounted for most culture-positive dialysis-related infections in both modalities. In HD bloodstream infections, *Staphylococcus aureus* was the most frequent isolate, whereas *Staphylococcus epidermidis* was most frequent among PD peritonitis episodes. Exact organism counts and percentages by modality and infection type are provided in Table 3.

**Table 3:** Microbiological profile of documented infections.

Microbiological variable	HD (*n* = 216)	PD (*n* = 51)	*p*-value
Culture-positive documented infection isolates, total	48 isolates	7 isolates	—
Gram-positive organisms, total	29 isolates (60.4% of isolates; 13.4% of cohort)	4 isolates (57.1% of isolates; 7.8% of cohort)	—
*Staphylococcus aureus*	14 isolates (29.2% of isolates; 6.5% of cohort)	1 isolate (14.3% of isolates; 2.0% of cohort)	—
*Staphylococcus epidermidis*	7 isolates (14.6% of isolates; 3.2% of cohort)	2 isolates (28.6% of isolates; 3.9% of cohort)	—
Other coagulase-negative Staphylococcus spp.	5 isolates (10.4% of isolates; 2.3% of cohort)	1 isolate (14.3% of isolates; 2.0% of cohort)	—
Other gram-positive organisms	3 isolates (6.3% of isolates; 1.4% of cohort)	0 (0.0%)	—
Gram-negative organisms, total	15 isolates (31.3% of isolates; 6.9% of cohort)	2 isolates (28.6% of isolates; 3.9% of cohort)	0.18
*Escherichia coli*	4 isolates (8.3% of isolates; 1.9% of cohort)	1 isolate (14.3% of isolates; 2.0% of cohort)	—
Klebsiella spp.	3 isolates (6.3% of isolates; 1.4% of cohort)	1 isolate (14.3% of isolates; 2.0% of cohort)	—
Pseudomonas spp./other gram-negative organisms	8 isolates (16.7% of isolates; 3.7% of cohort)	0 (0.0%)	—
Fungal organisms	4 isolates (8.3% of isolates; 1.9% of cohort)	1 isolate (14.3% of isolates; 2.0% of cohort)	0.44
*S. aureus*-caused BSI among HD BSI episodes	12/24 BSI episodes (50.0%)	Not applicable	—
*S. epidermidis*-caused BSI among HD BSI episodes	4/24 BSI episodes (16.7%)	Not applicable	—
Gram-negative-caused BSI among HD BSI episodes	4/24 BSI episodes (16.7%)	Not applicable	—
Fungal-caused BSI among HD BSI episodes	1/24 BSI episodes (4.2%)	Not applicable	—
*S. epidermidis*-caused peritonitis among PD peritonitis episodes	Not applicable	2/6 episodes (33.3%)	—
*S. aureus*-caused peritonitis among PD peritonitis episodes	Not applicable	1/6 episodes (16.7%)	—
Gram-negative-caused peritonitis among PD peritonitis episodes	Not applicable	2/6 episodes (33.3%)	—
Fungal-caused peritonitis among PD peritonitis episodes	Not applicable	1/6 episodes (16.7%)	—

Microbiological percentages are shown as both percentages of culture-positive isolates and percentages of the modality cohort when applicable. Organism-specific BSI and peritonitis percentages use infection-type denominators (HD BSI episodes *n* = 24; PD peritonitis episodes *n* = 6).

### Hospital visits and severity

Infection-related hospitalization was more frequent in HD patients (12.0%) than in PD patients (5.9%, *p* = 0.04; Table 4). The median number of all-cause hospitalizations was calculated across the full modality cohort, including patients with zero admissions, and was higher in HD patients (1 [IQR 0–2]) than in PD patients (0 [IQR 0–1], *p* = 0.01). All ICU admissions were nested within the infection-related hospitalization group; specifically, 9 of the 26 HD patients hospitalized for infection subsequently required ICU admission, and 1 of the 3 PD patients hospitalized for infection required ICU admission.

**Table 4:** Severity and clinical outcomes.

Outcome	HD (*n* = 216)	PD (*n* = 51)	*p*-value
Patients with > = 1 all-cause hospitalization	118 (54.6%)	20 (39.2%)	0.04
All-cause hospitalizations, median (IQR)	1 (0–2)	0 (0–1)	0.01
Infection-related hospitalization	26 (12.0%)	3 (5.9%)	0.04
ICU admission among infection-hospitalized patients	9 (4.2% cohort; 9/26, 34.6%)	1 (2.0% cohort; 1/3, 33.3%)	0.21
CRP (mg/L), median (IQR)	11.2 (6.5–18.4)	7.8 (4.1–12.3)	0.03
Severe sepsis	6 (2.8%)	1 (2.0%)	0.18
Mortality related to infection	4 (1.9%)	1 (2.0%)	0.39

ICU admissions were counted only among patients already hospitalized for infection. The median all-cause hospitalization value was calculated across the full cohort, including patients with no hospitalization, and is therefore not inconsistent with the presence of ICU admissions in a smaller severe-infection subgroup.

ICU admission therefore represents a severity subgroup among infection-related hospitalizations rather than a separate denominator or event category. ICU admission occurred in 9 HD patients (4.2% of the HD cohort; 34.6% of HD infection-related hospitalizations) and 1 PD patient (2.0% of the PD cohort; 33.3% of PD infection-related hospitalizations; *p* = 0.21). Severe sepsis was reported in 3% of HD patients and 1% of PD patients. Infection-related mortality was low in both groups (HD: 2%, PD: 1%).

### Laboratory parameters

**Figure 1:**
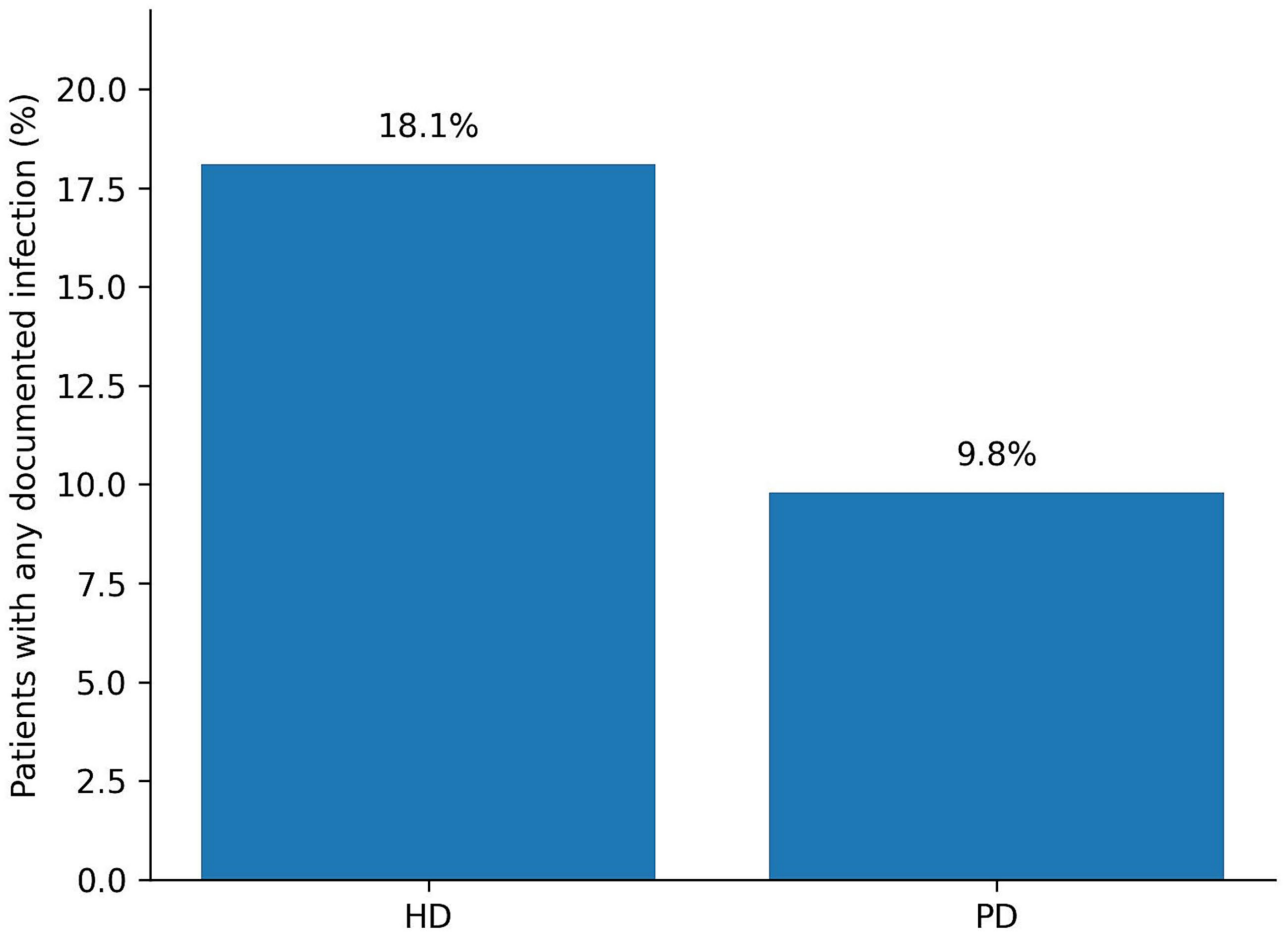
Infection rate comparison between HD and PD. Bar chart showing patient-level any documented infection rates: HD = 18.1% and PD = 9.8%.

Laboratory findings are summarized in Table 5. HD patients had lower hemoglobin levels and higher inflammatory markers. Median C-reactive protein (CRP) was significantly higher in HD patients (11.2 mg/L [IQR 6.5–18.4]) compared with PD patients (7.8 mg/L [IQR 4.1–12.3], *p* = 0.03). PD patients exhibited higher creatinine and uric acid levels. HD patients had significantly higher HbA1c values (7.8 ± 1.9% vs. 6.9 ± 1.6%, *p* < 0.001), while fasting blood sugar levels were comparable between groups (Figure 1).

**Table 5:** Laboratory parameters comparison.

Laboratory parameter	HD (*n* = 216)	PD (*n* = 51)	*p*-value
Urea (mg/dL)	15.8 ± 6.4	18.1 ± 7.2	0.01
Creatinine (μmol/L)	480 ± 210	690 ± 310	—
Sodium (mmol/L)	134.2 ± 3.1	135.1 ± 3.4	0.04
Potassium (mmol/L)	4.5 ± 0.8	4.2 ± 0.7	0.02
Uric acid (μmol/L)	280 ± 95	310 ± 110	0.03
HbA1c (%)	7.8 ± 1.9	6.9 ± 1.6	
FBS (mg/dL)	145 ± 52	132 ± 48	0.06
CRP (mg/L)	10.4 ± 6.8	7.1 ± 5.4	0.01

PD patients exhibited higher creatinine and uric acid levels. HD patients had significantly higher HbA1c values (7.8 ± 1.9% vs. 6.9 ± 1.6%, *p* < 0.001), while fasting blood sugar levels were comparable between groups.

### Paths to infection risk factors

Table 6 summarizes the multivariable analysis and HD treatment-frequency stratification. Diabetes mellitus and CVC use were strong predictors of infection in HD patients. HD frequency was also summarized descriptively: infection occurred in 37 of 207 patients receiving three sessions per week (17.9%) and 2 of 9 patients receiving four sessions per week (22.2%). Because only 9 patients received four sessions weekly for clinical indications, this subgroup was not treated as an independent predictor in the final model. In PD patients, catheter duration longer than 3 years was associated with increased infection risk. Elevated CRP and multiple comorbidities showed a dose–response relationship with infection risk across both modalities.

**Table 6:** Risk factors for infection (multivariate summary).

Risk factor/stratification	HD infection (%)	PD infection (%)	Association
Diabetes mellitus	Higher	Higher	Strong predictor
CVC access (HD)	29%	—	Strongest HD risk factor
AVF/AVG access (HD)	8%	—	Protective
HD frequency: three sessions/week	37/207 (17.9%)	—	Reference HD schedule
HD frequency: four sessions/week	2/9 (22.2%)	—	Descriptive only; not independently associated after adjustment because subgroup was small
PD catheter duration >3 years	—	18%	Significant
Age >65 years	Higher	Lower	Stronger effect in HD
CRP > 10 mg/L	Strong predictor	Moderate predictor	Inflammation-linked
Multiple comorbidities	Higher	Higher	Dose–response effect

### Yearly trends and figures

**Figure 2:**
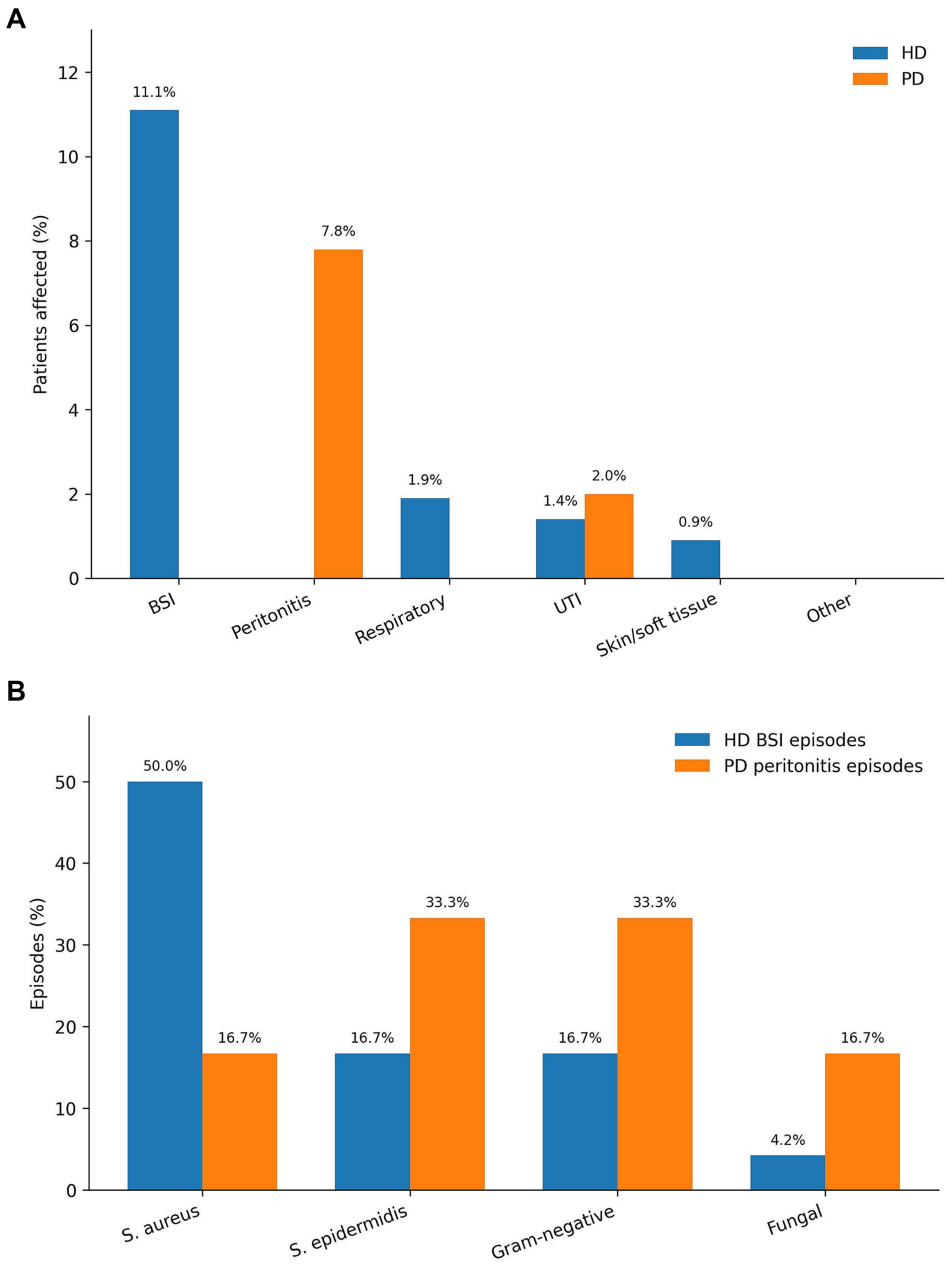
Distribution of infection types by dialysis modality. Panel **(A)** separates infection types from microbiology and shows dialysis-related, respiratory tract/pneumonia, urinary tract, skin/soft-tissue, and other infections by modality. Causative organisms within dominant dialysis-related infection types. Panel **(B)** shows causative organism distribution within HD bloodstream infection episodes and PD peritonitis episodes. *S. aureus* predominated among HD bloodstream infections, while *S. epidermidis* and gram-negative organisms were common among PD peritonitis episodes.

Infection rates declined modestly over the study period, from 20% in 2023 to 16% in 2025 among HD patients and from 11 to 9% among PD patients. Figure 2A summarizes infection types by modality, and Figure 2B summarizes the causative organisms within the dominant dialysis-related infection type for each modality.

## Discussion

In this report, we found clinically meaningful differences in dialysis-related infection patterns, severity, and outcomes between HD and PD patients over the 3-year period from 2023 through 2025. HD patients had a higher dialysis-related infection burden, mainly driven by bloodstream and access-related infections, whereas PD patients were primarily affected by peritonitis. Secondary non-dialysis infections were uncommon and did not drive the primary modality-specific comparison.

Because the study was conducted in a single Saudi tertiary dialysis center and the population was predominantly Saudi, the conclusions are framed for local and comparable Saudi dialysis populations. Differences in ethnicity, healthcare access, infection-control infrastructure, dialysis modality selection, and vascular-access practices may limit direct extrapolation to non-Saudi or multicenter international cohorts.

Quality improvement interventions can effectively reduce HD-associated infections when implemented consistently. A 2022 systematic review reported that multipronged interventions—including staff education, catheter care bundles, and audit-feedback mechanisms—reduced CRBSIs by up to 50% (16). However, the effectiveness of these interventions depends on sustained adherence, which remains challenging for many dialysis centers. The year-over-year decrease in HD infection rates observed in our study may reflect improved adherence to infection control practices, although further confirmation is needed.

Compared with HD patients, PD patients in our cohort had significantly lower overall infection rates but remained susceptible to peritonitis, consistent with global trends. The 2023 revised ISPD guidelines emphasize that exit-site and tunnel infections strongly predict peritonitis and catheter loss (3). Our findings support these recommendations, as PD catheter duration and exit-site condition significantly influenced infection risk. A 2024 systematic review reported increasing rates of nontuberculous mycobacterial infections in PD patients, reflecting evolving microbiological patterns in PD populations (17). Although these organisms were not predominant in our cohort, their emergence underscores the need for continued surveillance. Recent literature also highlights the importance of patient education, technique training, and home environment in preventing PD-related infections (18). This may explain the relatively stable infection rates in our PD cohort, as all patients undergo structured training and follow-up at our center. Nevertheless, the occurrence of peritonitis in some PD patients reinforces the need to promote aseptic technique and early recognition of infection.

Hospitalization patterns in our cohort mirrored global data. HD patients were hospitalized more frequently and experienced more severe infection outcomes—including ICU admissions—than PD patients. Infection was identified as a leading cause of hospitalization and mortality among HD patients in a 2023 review of HD complications (19). Similarly, the 2024 APIC guideline revision described frequent healthcare contact and environmental exposure among HD patients as unique factors contributing to infection control challenges (20). This may explain the increased severity of infections observed in our HD sample.

Laboratory findings in our study supported the clinical observations. HD patients had elevated CRP levels, consistent with chronic inflammation associated with vascular access manipulation and extracorporeal circuit use. Recent nephrology literature reports that elevated inflammatory markers increase infection risk and worsen outcomes in HD patients (21). PD patients, on the other hand, often exhibit higher creatinine and uric acid levels than HD patients; differences in solute clearance and residual renal function may contribute to infection susceptibility (22).

Our results suggest that infection prevention strategies must be modality-specific. For HD, minimizing catheter use, increasing AVF maturation rates, and implementing strict catheter-care bundles remain essential (23). For PD, prevention and early identification of exit-site infections (24) and continued patient-centered education are critical, consistent with ISPD guidance. Emerging research also highlights the importance of antimicrobial stewardship in dialysis care to reduce resistant infections (25).

The gradual decline in infection rates across both modalities over time is encouraging and may reflect improvements in infection control practices, staff training, and surveillance. National surveillance programs have reported similar trends, showing that reductions in dialysis-related infections are associated with robust quality-improvement initiatives and standardized reporting systems (26–29).

### Strengths

One of the significant strengths of this study was the provision of a large well-characterized cohort (N = 267 ESRD patients) studied closely over a three-year period, which provided a comprehensive comparison of hemodialysis with peritoneal dialysis in a well-defined setting. This study was a result of robust research in terms of the demographic, clinical, laboratory, microbiological, and hospitalization aspects and data had been collected and able to provide a broad perspective on the global infection burden and the possible sequelae. Another important feature in the review was the inclusion of standard diagnosis for infections in our study (CDC/NHSN and ISPD), which allowed uniform interpretation with international data and allowed for meaningful global comparison. The potential study of how trends in infection patterns evolved over three straight years would add an important temporal trend dimension to the study.

### Limitations

There are several limitations that need to be considered. A retrospective design includes built-in risks of missing data, variability in documentation, and misclassification of infections. This study was conducted in a single tertiary institution and included a predominantly Saudi population, limiting generalizability to other geographic regions, healthcare systems, or non-Saudi dialysis populations. Microbiological data were drawn from routinely obtained clinical samples and may underestimate infections due to fastidious or uncultured organisms. Antibiotic exposure was not included as an independent analytic variable because timing, dose, indication, and duration were incompletely standardized in the retrospective records. Viral infections were not systematically assessed because virology testing was not uniformly available across the whole cohort. Hemodiafiltration was not analyzed separately from conventional HD, and patients who permanently changed dialysis modality were excluded; therefore, the findings should be interpreted as modality-specific associations within stable HD and PD treatment groups.

## Conclusion

This study showed that infections remain an important clinical problem in a predominantly Saudi cohort of patients receiving HD or PD at a tertiary center in Hail. HD patients experienced greater infection-related healthcare use, largely related to catheter exposure and comorbidities such as diabetes mellitus, whereas peritonitis remained the key concern in PD. These findings support Saudi-context, modality-specific prevention, surveillance, and targeted interventions to reduce infection-related harm and improve patient outcomes.

### Recommendations

Hemodialysis units should minimize catheter dependence through timely creation of AVF and strict compliance with catheter-care bundles. Ongoing staff education, competence evaluation and assessment, and compliance monitoring is crucial to reinforce infection-prevention interventions. Focus should be given to structured patient education, early detection of exit-site infections, and the reinforcement of aseptic technique for peritoneal dialysis. Better surveillance approaches, antimicrobial stewardship and coordination among nephrology, infection control, and nursing teams may enhance success in both approaches. The direction for further research is, however, to explore molecular epidemiology, antimicrobial-resistance trends, and the effectiveness of targeted quality-improvement interventions.

## Data Availability

The datasets presented in this study can be found in online repositories. The names of the repository/repositories and accession number(s) can be found in the article/Supplementary material.
