# Correction: Infectious disease outcomes in end-stage renal disease: the influence of dialysis type on patient vulnerability in Hail region

**DOI:** 10.3389/fmed.2026.1925755

**Published:** 2026-07-17

**Authors:** Rihab Akasha, Naif K. Binsaleh, Randa Abdeen Husien Abdalla, Obey Suliman, Ibrahim Nasser Abuqurayn, Sultan Alouffi

**Affiliations:** 1 Department of Medical Laboratory Science, College of Applied Medical Sciences, University of Hail, Hail, Saudi Arabia; 2 Medical and Diagnostic Research Centre, University of Hail, Hail, Saudi Arabia; 3 Nephrology Consultant, King Khalid Hospital, Hail, Saudi Arabia; 4 Nephrology Department Supervisor, Hail Cluster, Hail, Saudi Arabia

**Keywords:** catheter-related bloodstream infection, dialysis outcomes, hemodialysis, infection, peritoneal dialysis, peritonitis

Author Ibrahim Nasser Abuqurayn was erroneously spelled as Ibrahim Nasser Abuqurany.

Affiliation “Nephrology Consultant, King Khalid Hospital, Hail, Saudi Arabia” was erroneously given as “King Khalid Hospital, Hail, Saudi Arabia”.

Author “Randa Abdeen Husien Abdalla” was erroneously assigned to affiliation “Department of Clinical Laboratory Sciences, College of Applied Medical Sciences, Hail University, Hail, Saudi Arabia”. This affiliation has now been removed from the article.

Affiliation “Department of Medical Laboratory Science, College of Applied Medical Sciences, University of Hail, Hail, Saudi Arabia” was erroneously omitted for author “Randa Abdeen Husien Abdalla”.

Affiliation “Medical and Diagnostic Research Centre, University of Hail, Hail, Saudi Arabia” was erroneously omitted for authors “Rihab Akasha, Randa Abdeen Husien Abdalla and Sultan Alouffi”.

An incorrect **Funding** statement was provided:

“The author(s) declared that financial support was received for this work and/or its publication. This research has been funded by Science Research Deanship at University of Hail, Saudi Arabia through project number RCP-24108.”

The correct **Funding** statement reads:

“The author(s) declared that financial support was received for this work and/or its publication. This research has been funded by Scientific Research Deanship at University of Hail, Saudi Arabia through project number RCP-24 105.”

In the published article, there was a mistake in the **Acknowledgments** statement. The project number was displayed as RCP-24 108.

The correct **Acknowledgments** statement is: All authors express gratitude to the Scientific Research Deanship at University of Ha'il for their funding through the project number RCP-24 105.

In the **Acknowledgements**, an incorrect funding number was provided. The correct **Acknowledgement** reads:

“All authors express gratitude to the Scientific Research Deanship at University of Ha'il for their funding through the project number RCP-24 105.”

The original version of this article has been updated.

